# FXR agonist obeticholic acid reduces hepatic inflammation and fibrosis in a rat model of toxic cirrhosis

**DOI:** 10.1038/srep33453

**Published:** 2016-09-16

**Authors:** Len Verbeke, Inge Mannaerts, Robert Schierwagen, Olivier Govaere, Sabine Klein, Ingrid Vander Elst, Petra Windmolders, Ricard Farre, Mathias Wenes, Massimiliano Mazzone, Frederik Nevens, Leo A. van Grunsven, Jonel Trebicka, Wim Laleman

**Affiliations:** 1 Division of Liver and Biliopancreatic disorders, University Hospitals Leuven, KU Leuven - University of Leuven, Leuven, Belgium; 2 Liver Cell Biology Laboratory, Vrije Universiteit Brussel, Brussels, Belgium; 3 Department of Internal Medicine I, University of Bonn, Bonn, Germany; 4 Translational Cell and Tissue Research, Department of Imaging and Pathology, University Hospitals Leuven, KU Leuven - University of Leuven, Leuven, Belgium; 5 Translational Research Center for Gastrointestinal Disorders, KU Leuven - University of Leuven, Leuven, Belgium; 6 Centro de Investigación Biomédica en Red de Enfermedades Hepáticas y Digestivas (CIBERehd), Instituto de Salud Carlos II, Barcelona, Spain; 7 Laboratory of Molecular Oncology and Angiogenesis, Vesalius Research Center, Department of Oncology, KU Leuven - University of Leuven, Belgium; 8 Laboratory of Molecular Oncology and Angiogenesis, Department of Oncology, VIB, Leuven, Belgium

## Abstract

Hepatic inflammation drives hepatic stellate cells (HSC), resulting in liver fibrosis. The Farnesoid-X receptor (FXR) antagonizes inflammation through NF-κB inhibition. We investigated preventive and therapeutic effects of FXR agonist obeticholic acid (OCA) on hepatic inflammation and fibrosis in toxic cirrhotic rats. Cirrhosis was induced by thioacetamide (TAA) intoxication. OCA was given during or after intoxication with vehicle-treated rats as controls. At sacrifice, fibrosis, hemodynamic and biochemical parameters were assessed. HSC activation, cell turn-over, hepatic NF-κB activation, pro-inflammatory and pro-fibrotic cytokines were determined. The effect of OCA was further evaluated in isolated HSC, Kupffer cells, hepatocytes and liver sinusoidal endothelial cells (LSEC). OCA decreased hepatic inflammation and fibrogenesis during TAA-administration and reversed fibrosis in established cirrhosis. Portal pressure decreased through reduced intrahepatic vascular resistance. This was paralleled by decreased expression of pro-fibrotic cytokines (transforming growth-factor β, connective tissue growth factor, platelet-derived growth factor β-receptor) as well as markers of hepatic cell turn-over, by blunting effects of pro-inflammatory cytokines (e.g. monocyte chemo-attractant protein-1). *In vitro*, OCA inhibited both LSEC and Kupffer cell activation; while HSC remained unaffected. This related to NF-κB inhibition via up-regulated IκBα. In conclusion, OCA inhibits hepatic inflammation in toxic cirrhotic rats resulting in decreased HSC activation and fibrosis.

Conditions of sustained hepatic injury are associated with increasing hepatocyte loss and scar formation, resulting in progressive accumulation of fibrosis within the liver parenchyma^1^. The main cellular driver of fibrosis in this process has been shown to be the hepatic stellate cell (HSC)^2^. HSC are in turn incited by stimulated Kupffer cells (KC) and liver sinusoidal endothelial cells (LSEC) via paracrine production of pro-inflammatory and pro-fibrotic cytokines such as connective tissue growth factor (CTGF), transforming growth factor-β (TGF-β) and platelet-derived growth factor (PDGF). Under these conditions, HSC proliferate and transdifferentiate into myofibroblasts, deposing collagen type I and III as well as fibronectin in the hepatic extracellular matrix (ECM), eventually producing pro-fibrotic cytokines also in an autocrine fashion^3^. The development of hepatic fibrosis, and ultimately cirrhosis, is considered detrimental in the natural evolution of chronic liver disease as it interferes with the normal hepatic metabolic function and hampers hepatic portal blood flow which increases intrahepatic vascular resistance (IHVR), and ultimately leads to portal hypertension and its complications^4^.

For these reasons, interfering with the process of fibrogenesis has become an appealing therapeutic target for future treatment of chronic liver disease; especially since for many prevalent liver disorders such as primary biliary cirrhosis (PBC) and non-alcoholic steatohepatitis (NASH), treatment of the underlying liver disease remains extremely challenging^5^,^6^,^7^,^8^,^9^. From this perspective, the farnesoid-X receptor (FXR) appears a promising target. This nuclear receptor, which is essentially a bile-acid responsive transcription regulator of bile and lipid metabolism, has recently also been correlated with the development of hepatic fibrosis^10^,^11^,^12^. As an example hereof, FXR knock-out mice over time display increased hepatic inflammation and fibrosis^13^,^14^. Furthermore, the activation of FXR by means of highly potent and selective FXR agonists such as obeticholic acid (OCA) has been suggested to reduce fibrosis in two experimental models of *early-stage* liver fibrosis either by 2 weeks bile-duct ligation or after 12 weeks of treatment with porcine serum^12^. Contrarily, Fickert *et al.* have shown that after FXR-knock-out, decreased hepatic FXR expression is protective of fibrosis progression in experimental cholestasis, while it is totally unrelated to fibrogenesis under non-cholestatic conditions such as carbon tetrachloride (CCl_4_) or S. mansoni-infection^15^. Furthermore, we and others were unable to detect direct effects of FXR stimulation on human and rodent HSC activation and hypercontractility *in vitro*^15^,^16^. Conceptually however, the FXR pathway might also decelerate fibrogenesis indirectly by targeting hepatic inflammation since it has been shown to reciprocally counter-balance nuclear factor-kappa beta (NF-κB) mediated- transcription of pro-inflammatory cytokines in a variety of cell types^13^,^17^,^18^.

Given this background and controversies, we aimed to explore, both prophylactically as therapeutically, the effects of FXR-agonism by means of OCA, an oral first-in-class FXR agonist, on hepatic inflammation and fibrosis in a model of toxic non-cholestatic cirrhosis by means of thioacetamide ingestion in rats and to correlate these findings to different cytokines and parenchymal and non-parenchymal cell types exposed to FXR-agonism *in vitro*.

## Materials and Methods

### Animals

60 Male Wistar rats (Janvier, France) weighing 200–250 grams were randomly allocated to 4 experimental groups (Supplementary Figure S1). In all animals, thioacetamide was used as hepatoxin, which was validated earlier^19^ to induce histology-proven cirrhosis after 18 weeks of administration in drinking water. In a first, prophylactic arm of the study, either vehicle (group 1, TAA) or 10 mg/kg of the FXR agonist OCA (group 2, TAA + OCA) was administered by gavage every 2 days during the last 4 weeks of the TAA intoxication protocol (thus in the last phase of advanced fibrosis/cirrhogenesis). In a second, therapeutic arm of the study, again either vehicle (group 3, TAA + 4) or 10 mg/kg of the FXR agonist OCA (group 4, TAA + 4 + OCA) was administered through gavage every 2 days for 4 weeks after stopping the 18 week-regimen of TAA intoxication (thus after obtaining cirrhosis). Parenchymal liver cells were isolated from Balb/cByJ mice (25–35 g, Charles River Laboratories, France). Approval for all experiments was obtained from the KULeuven Animal Ethics Committee and all experiments were conducted according to the approved guidelines.

### *In vivo* hemodynamic and biochemical parameters

At sacrifice, portal pressure (PP), mean arterial pressure (MAP) and mesenteric blood flow (MBF) were measured as described^16^. Samples were collected by aortic puncture in heparinized tubes and analyzed for routine plasma hepatic enzymes by automated procedure^19^. Hepatic tissue samples were snap-frozen for molecular analysis.

### Isolation of mouse liver cells and *in vitro* stimulation experiments

HSCs, liver sinusoidal endothelial cells (LSEC) and hepatocytes (HEP) were isolated for mouse liver and cultured according to validated protocols^20^,^21^. Kupffer cells (KC) were isolated by fluorescence-activated cell sorting after collagenase/pronase perfusion, using an F4/80 antibody (Invitrogen, USA). Cell purity and functionality were confirmed on morphology and by quantitative polymerase chain reaction (qPCR) for following marker genes (Supplementary Methods S2): Cyp3a11 (HEP), CD32b (LSEC), desmin and Acta2 (HSC)^22^. 2 Hours after isolation (4 hours for HEP), cells were washed and either solvent or OCA was added to the medium at concentrations of 0.1, 1 and 10 μM, together with vehicle, 1 mg/mL TGF-β1 (R&D Systems, Wiesbaden-Nordenstadt, Germany), TNF-α or LPS. All cells were collected for molecular analysis 24 h after incubation, except for culture-activated HSC that were further stimulated for 7 days. LX2 cells were provided by Vijay H. Shah (Mayo Clinic, Rochester, NY), originally established by Scott Friedman^23^.

### Assessment of hepatic fibrosis by image analysis

Fresh liver samples were fixed in formaldehyde 6%, paraffin embedded, sectioned and Sirius-red stained. Areas of fibrosis were detected and scored with automated color threshold image analysis by use of an Olympus BX60 microscope and Stream essentials software (version 1.9 [2013]; Olympus, Belgium). Slides from both the left lateral and the middle hepatic lobe were then evaluated per rat and blinded to the investigator analyzing the samples. 10 consecutive image-centered portal tracts were scored per rat for fibrosis and were represented as a percentage of the total liver parenchyma at 10X magnification.

### Caspase-3 Immunohistochemistry

5 μm-thick frozen tissue samples were stained using the BondTM Polymer Refine Red Detection kit on the Bond Max autostainer (Leica). Primary antibody was directed against cleaved Caspase-3 (1/100, Cell Signaling). Positive cells were quantified in five higher power fields.

### Hepatic hydroxyproline content

Three corresponding segments (200 mg) from the middle hepatic lobe were hydrolyzed in HCl (6N), filtered and incubated with chloramine T (2.5 mM) and Ehrlich’s reagent^7^. The hepatic hydroxyproline content was determined photometrically in the acquired liver hydrolysates by absorption measurements at 558 nm. Results are expressed as μg/g of wet liver tissue.

### RT-PCR

Supplementary Methods S3.

### Western blot

Supplementary Methods S4.

### NF-κB activity assay

A liver specimen of about 100 mg was lysed in 1,5 ml RIPA buffer (50 mM Tris pH8, 150 mM NaCl, 0,5% SDS, 1% triton, 5 mM EDTA, 0,5% Na-deoxycholate) containing protease and phosphatase inhibitors (Roche, Belgium), and using Lysing matrix D (MP-biomedicals, USA) and a Ribolyser (Bio-rad, Belgium) 3 × 60 s at maximum speed. Protein concentration was determined using the Pierce BCA Protein Assay Kit (Thermo Scientific, USA). 30 μg of protein was used to determine NF-kB activity using the Active Motif (Belgium) p65-TransAM kit following the manufacturers’ protocol.

### *In situ*-liver perfusion

To evaluate total intrahepatic vascular resistance (IHVR), a flow-controlled perfusion study of the liver was performed with a continuously oxygenated and heated Krebs buffer solution as described^16^,^19^,^24^. After a 20 minute stabilization period, flow rates were increased by 5 ml/min every each 5 minutes starting at a 30 ml/min flow rate. Subsequent elevations in perfusion pressure were assessed at the level of the portal inflow catheter and as such reflective of total IHVR.

### Statistics

Data were all normally distributed. As such, data were expressed as means ± standard error of means (SEM) and subjected to parametrical statistics. Comparing two unpaired groups, the Student t test was applied; for comparison of multiple groups, a one-way analysis of variance was applied. If positive, a subsequent pair-wise comparison was performed by means of Bonferroni t-testing. Correlations were assessed by linear regression analysis. P-values below 0.05 were considered statistically significant. Statistical analysis was performed by means of GraphPad Prism software (version 6.02[2013]; GraphPad Software Inc., USA).

## Results

### FXR agonist OCA reduces fibrosis in TAA cirrhotic rats

**Figure 1:**
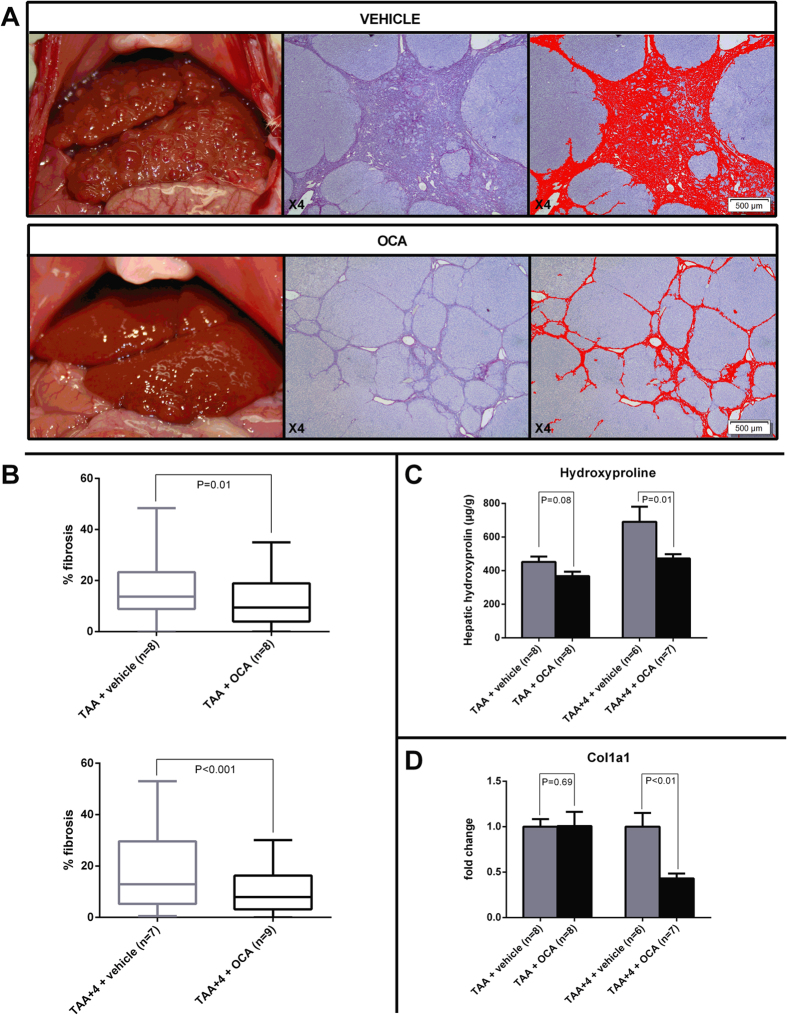
Effects of FXR agonist OCA on fibrosis in TAA rats. (**A**) Representative macroscopic and microscopic (Sirius red stain with subsequent image analysis) illustration of a vehicle- vs. OCA-treated rat in the therapeutic arm of the study. (**B**) Image analysis data showing a decreased detection of fibrosis in both the prophylactic and the therapeutic study groups (n = 7–9 animals per group). (**C**) Following OCA treatment, hepatic hydroxyproline content is significantly decreased in the therapeutic study arm and nearly significant in the prophylactic arm of the study, confirming decreased hepatic fibrosis (n = 6–8 animals per group). (**D**) In the therapeutic study, this is associated with a decreased hepatic expression of col1a1 mRNA (n = 6–8 animals per group). FXR, Farnesoid-X receptor; TAA, thioacetamide; col1a1, collagen type I.

**Figure 2:**
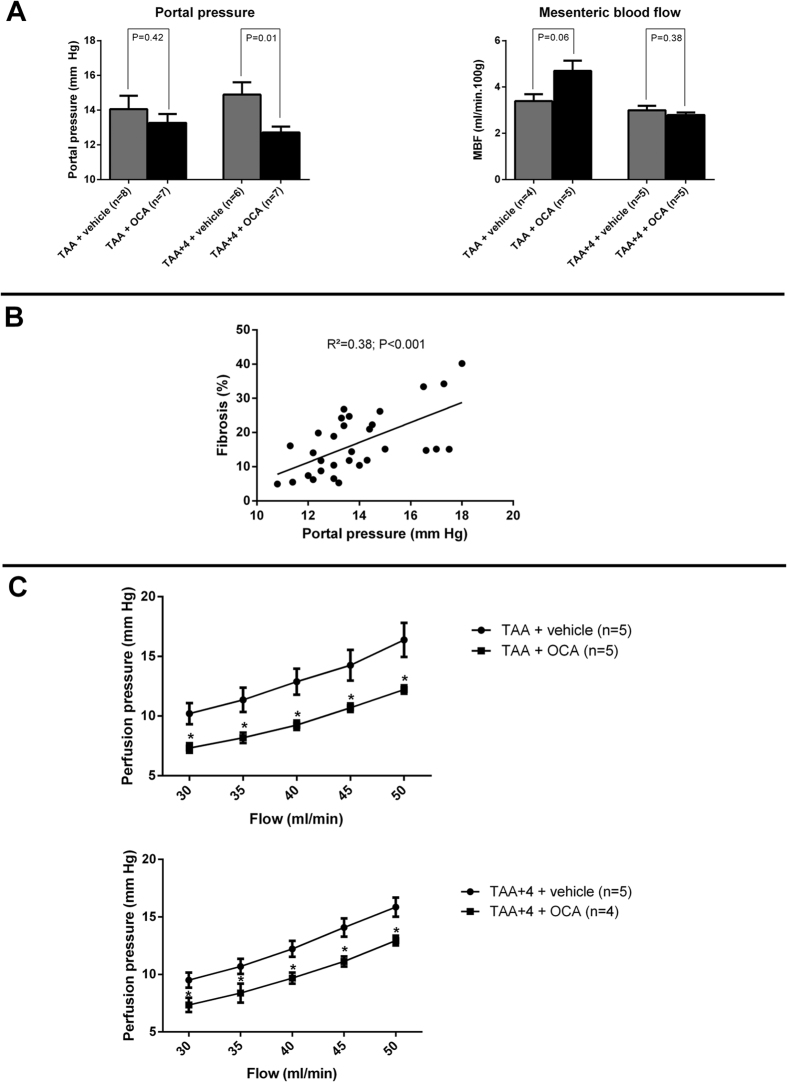
Hemodynamic parameters and intrahepatic vascular resistance. (**A**) Portal pressure is decreased in TAA cirrhotic rats (therapeutic study arm) following 4 weeks of OCA treatment, without affecting mesenteric blood flow (n = 6–8 and 4–5 rats per groups, respectively). (**B**) Portal pressure is highly and significantly correlated with the amount of hepatic fibrosis, as assessed by image analysis in rats from all groups, regardless of OCA treatment. (**C**) *In-situ* liver perfusion data showing decreased perfusion pressure at each flow rate in both the preventive and therapeutic study arm, indicative of a decreased intrahepatic vascular resistance. TAA, thioacetamide; OCA, obeticholic acid; MBF, mesenteric blood flow.

After 18 weeks of TAA intoxication, all vehicle-treated animals had developed cirrhosis, as confirmed by Sirius-red-stained liver histology (Fig. 1A). There was no difference in mortality between OCA-treated and -untreated animals (88% overall survival). Physical and biochemical variables amongst different groups are given in Table 1. A clear decrease in plasma alanine aminotransferase (ALT) levels was observed after OCA treatment in the therapeutic study arm (97 ± 4 vs. 79 ± 4 IU/L in TAA + 4 vs. TAA + 4 + OCA, n = 6 and 9 resp.; P = 0.01; Table 1). Upon image analysis, the amount of fibrosis is clearly decreased in both the prophylactic (16.5 ± 1.2 vs. 12.2 ± 1.1% in TAA vs. TAA + OCA; n = 8 in both groups; P = 0.01; Fig. 2B) and the therapeutic (17.1 ± 1.7 vs. 10.3 ± 0.9% in TAA + 4 vs. TAA + 4 + OCA; n = 7 and 9 resp.; P < 0.001; Fig. 1B) study groups upon OCA treatment. The latter is confirmed by a decrease in hepatic hydroxyproline content (691 ± 91 vs. 473 ± 24 in TAA + 4 vs. TAA + 4 + OCA; n = 6 and 7 resp.; P = 0.01; Fig. 1C) and a decreased hepatic collagen type I mRNA expression (Fig. 1D), (Fig. 1A–D, Table 1).

### The reduction in hepatic fibrosis by OCA is associated with a decreased portal pressure through reduced intrahepatic vascular resistance

Upon treatment with OCA, portal pressure is significantly decreased in the therapeutic study arm (14.9 ± 0.7 vs. 12.7 ± 0.3 mm Hg in TAA + 4 vs. TAA + 4 + OCA; n = 6 and 7 resp.; P = 0.01; Fig. 2A), while mesenteric blood flow was elevated as expected given the phenomenon of splanchnic hyperemia, it remained unaffected by OCA gavage in both groups. Interestingly, the overall degree of fibrosis correlated nicely with the degree of portal hypertension in cirrhotic rats (R^2^ = 0.38; P < 0.001; Fig. 2B). Upon liver perfusion, overall perfusion pressures were markedly lower for any given flow following OCA treatment in both the prophylactic and the therapeutic study arm at any flow rate, indicative of a decreased total IHVR (1.05 ± 0.08 vs. 0.78 ± 0.05 and 1.22 ± 0.11 vs. 0.99 ± 0.05 mm Hg*min*100 g/ml in TAA vs. TAA + OCA and TAA + 4 vs. TAA + 4 + OCA; n = 5 in all groups except TAA + 4 + OCA: n = 4; P < 0.001; Fig. 2C), (Fig. 2A–C).

### In cirrhotic rat livers, OCA decreases markers of HSC activation, markers of apoptosis and proliferation, and also the expression of pro-inflammatory and pro-fibrotic cytokines

**Figure 3:**
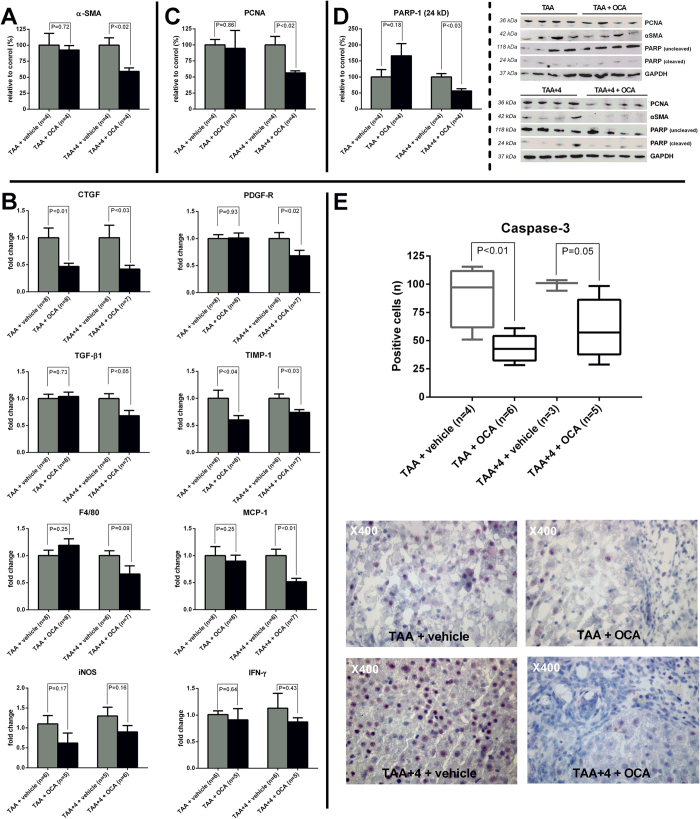
Hepatic levels of HSC activation marker α-SMA and cytokines involved in fibrogenesis, fibrinolysis, inflammation, proliferation and apoptosis. (**A**) In the therapeutic study arm, the hepatic protein levels of α-SMA, assessed by Western blot, are clearly decreased following OCA treatment indicative of decreased HSC activation (n = 4 per group). (**B**) RT-PCR data showing decreased expression of pro-fibrotic (CTFG, PDGF-R, TGF-β1), anti-fibrinolytic (TIMP-1) as well as pro-inflammatory cytokines in either the therapeutic study arm alone or both upon OCA treatment (n = 6–8 per group). (**C**) Western blot data showing decreased levels of proliferation marker PCNA in OCA treated rats from the therapeutic study arm. (**D**) Western blot data showing a decrease in the small degradation fragment (24 kD) of PARP-1 in OCA treated rats from the therapeutic study arm, indicative of decreased capase-3 activity. (**E**) Immunohistochemical staining for caspase-3 showing decreased caspase-3 positivity in both the prophylactic and therapeutic study arm. HSC, hepatic stellate cell; α-SMA, alpha-smooth muscle actin; OCA, obeticholic acid; RT-PCR, reverse-transcriptase polymerase chain reaction; CTGF, connective tissue growth factor; PDGF-R, platelet-derived growth factor-beta receptor; TGF-β1, Transforming growth factor-β1; TIMP-1, tissue inhibitor of metallopeptidase-1; MCP, monocyte chemo-attractant protein-1; iNOS, inducible nitric oxide synthase; IFN-γ, interferon-gamma; PCNA, proliferating cell nuclear antigen; PARP-1, poly ADP ribose polymerase-1.

In the therapeutic study arm, a significant decreased hepatic protein level of alpha-smooth muscle actin (α-SMA) was observed on Western blot, indicative of decreased HSC activation (P < 0.02; Fig. 3A). In accordance with these findings, the hepatic expression of connective tissue growth factor (CTGF), one of the main pro-fibrotic cytokines driving hepatic fibrogenesis, was reduced in both arms (P < 0.03; Fig. 3B). In the therapeutic study arm, this was further associated with a decreased expression of both transforming growth factor-β1 (TGF-β1) and platelet-derived growth factor-beta receptor (PDGFβ-R) (P < 0.05 and 0.02 resp., Fig. 3B). Additionally, the expression of tissue inhibitor of metallopeptidase-1 (TIMP-1), a known inhibitor of metalloproteinases and thus ECM degradation, appears down-regulated in both study arms (P < 0.04; Fig. 3B). At the protein level, proliferating cell nuclear antigen (PCNA), a validated global marker of pro-inflammatory and pro-fibrotic cell proliferation^7^, was decreased in cirrhotic rats following OCA treatment (P < 0.02; Fig. 3C). Also poly ADP ribose polymerase-1 (PARP-1), known substrate of active caspase-3 and thus a marker of apoptosis, displays decreased cleavage upon OCA exposure, illustrated by a decrease in the small (24 kD) cleaving fragment compared to the large uncleaved PARP-1 protein (118 kD) (P < 0.03; Fig. 3D). The latter was confirmed by a clear decrease of immunohistochemical staining positivity for caspase-3 in both the prophylactic and the therapeutic study group (P ≤ 0.05; Fig. 3E), (Fig. 3A–E).

### OCA does not directly affect either rodent or human HSC *in vitro*

**Figure 4:**
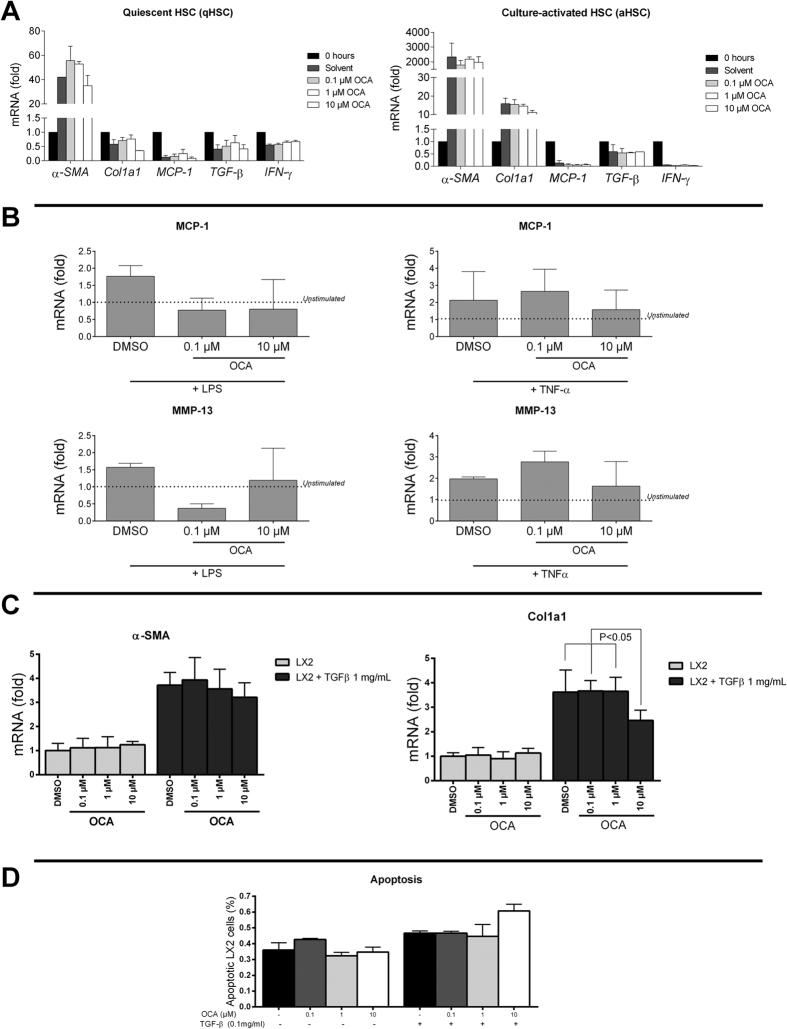
*In vitro* response to OCA in both quiescent and activated, both primary murine and human LX2 HSC lines. (**A**) The expression of pro-inflammatory and pro-fibrotic cytokines is unaltered in both quiescent freshly collected, as well as 7-day culture-activated HSC upon stimulation *in vitro* with 0.1–10 μM OCA. (**B**) OCA does not alter the expression of MCP-1 or MMP-13 in primary HSC stimulated with LPS or TNF-α. (**C**) In the human HSC line LX2, stimulation with 1 mg/mL TGF-β1 induces the expression of α-SMA and col1a1, which cannot be prevented by co-stimulation with OCA; with the exception of col1a1 expression at 10 μM OCA. (**D**) OCA does not affect the *in vitro* fraction of apoptotic LX2 cells, neither in basic nor 0.1 mg/ml TGF-β stimulated conditions. FXR, Farnesoid-X receptor; SHP, small heterodimer partner; α-SMA, alpha-smooth muscle actin; qHSC, quiescent hepatic stellate cell, aHSC, activated hepatic stellate cell; OCA, obeticholic acid; RT-PCR, reverse-transcriptase polymerase chain reaction; col1a1, collagen type I; MCP-1, monocyte chemo-attractant protein-1; PDGF-R, platelet-derived growth factor-beta receptor; TGF-β, transforming growth factor-β; IFN-γ, interferon-gamma; MMP-13, matrix metallopeptidase 13; TNF-α, tumor necrosis factor-alpha; LPS, lipopolysaccharides.

In freshly isolated, quiescent primary HSC, the *in vitro* expression of pro-inflammatory and pro-fibrotic cytokines was not significantly affected by stimulation with 0.1–10 μM OCA (Fig. 4A). While HSC displayed clearly activated phenotype after 7 days of culture-growth, with a 2000-fold increase in α-SMA expression, they remained unaffected by OCA (Fig. 4A). Also upon *in vitro* stimulation with tumor necrosis factor-alpha (TNF-α) or lipopolysaccharides (LPS), primary HSC remained unresponsive to OCA (Fig. 4B). In the human hepatic stellate cell line LX2^23^, activation was seen following *in vitro* stimulation with 1 mg/mL transforming growth factor-beta1 (TFB-β1) with increased expression of α-SMA and col1a1. This was unaltered by simultaneous incubation with 0.1–10 μM OCA, with the exception of slightly decreased col1a1 expression in TGF-β1 stimulated LX2 cells at the highest concentration of OCA 10 μM (P ≤ 0.05; Fig. 4C). Finally, OCA failed to affect apoptosis amongst LX2 cells *in vitro*, neither in the presence nor absence of TGF-β1 (Fig. 4D), (Fig. 4A–D).

### OCA prevents the *in vitro* activation of Kupffer cells (KC) and liver sinusoidal endothelial cells (LSEC) by LPS or TNF-α

**Figure 5:**
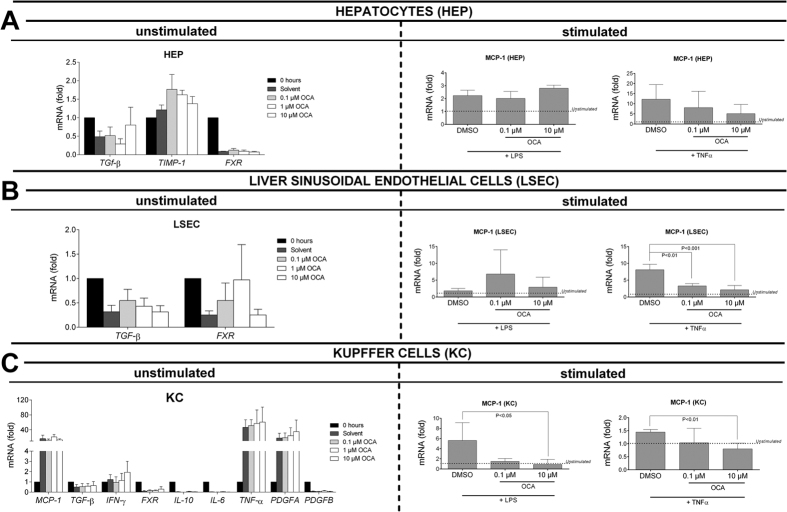
Effect of OCA on primary hepatocytes (HEP), liver sinusoidal endothelial cells (LSEC) and Kupffer cells (KC), in basal or LPS-/TNF-α-stimulated conditions. (**A**) The expression of MCP-1 is increased in hepatocytes following LPS- or TNF-α stimulation, yet remains unaffected by co-stimulation with OCA. (**B**) While in unstimulated LSEC, OCA does not affect the expression of pro-fibrotic cytokines such as TGF-β, it clearly prevents the increased expression of MCP-1 following *in vitro* stimulation with TNF-α. (**C**) While in unstimulated KC, OCA does not affect the expression of pro-inflammatory and pro-fibrotic cytokines; the expression of MCP-1 is decreased following co-incubation with 10 μM OCA. DMSO, dimethyl sulfoxide, vehicle; OCA, obeticholic acid; MCP-1, monocyte chemo-attractant protein-1; LPS, lipopolysaccharides; TGF-β, transforming growth factor-β; TIMP-1, tissue inhibitor of metallopeptidase-1; FXR, Farnesoid-X receptor; IFN-γ, interferon-gamma; IL-10, interleukin-10; IL-6, interleukin-6; PDGF, platelet-derived growth factor.

Primary hepatocytes (HEP) respond to LPS and especially TNF-α *in vitro* with increased expression of MCP-1, yet this remained unaffected by co-incubation with 0.1–10 μM OCA (Fig. 5A). While OCA failed to affect the expression of pro-inflammatory and pro-fibrotic cytokines in unstimulated LSEC and KC, it prevented the increased expression of MCP-1 following stimulation with TNF-α and LPS or TNF-α alone in LSEC and KC respectively (P < 0.05; Fig. 5B,C), (Fig. 5A–C).

### Reduced hepatic fibrosis by OCA is associated with decreased hepatic NF-κB pathway activity through up-regulated IκBα

**Figure 6:**
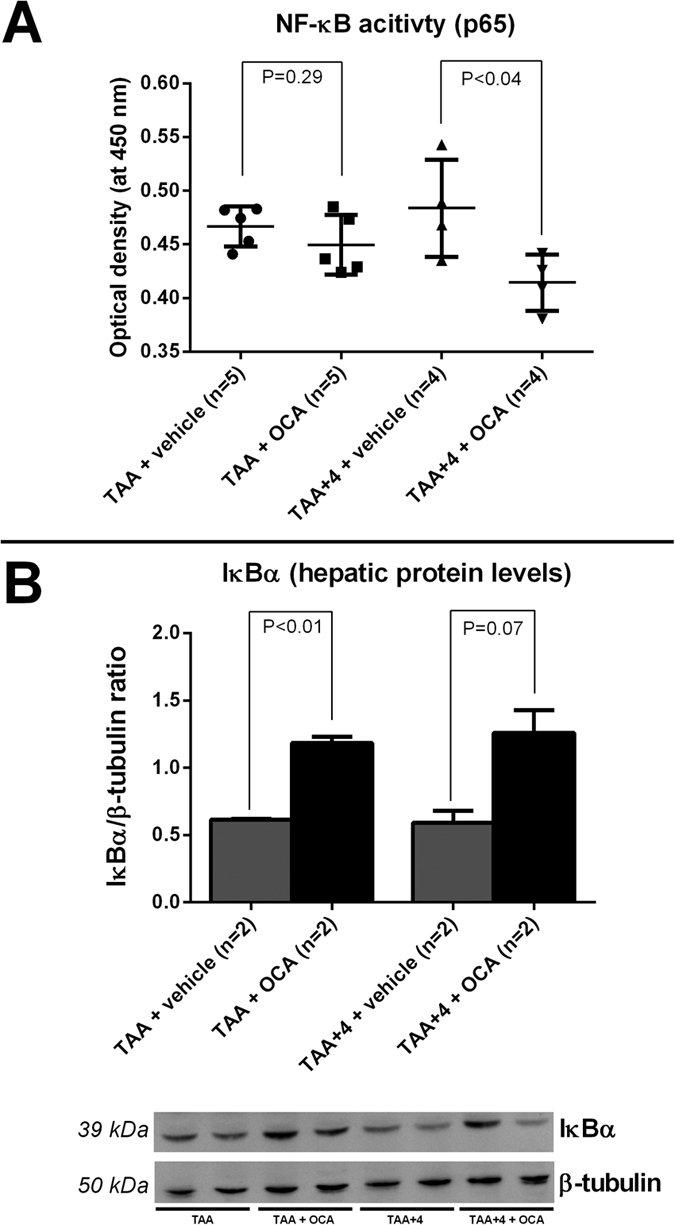
Hepatic NF-κB pathway activity in prophylactic and therapeutic study groups. (**A)** TransAM^®^ assay showing decreased p65 activation indicative of NF-κB pathway activity following OCA treatment in the therapeutic study arm. (**B**) IκBα is increased significantly after OCA administration in the prophylactic study arm. NF-κB, nuclear transcription factor-kappa beta; TAA, thioacetamide; OCA, obeticholic acid; IκBα, nuclear factor of kappa light polypeptide gene enhancer in B-cells inhibitor, alpha.

Upon treatment with OCA, decreased p65 (RelA) activation was indicative for decreased hepatic NF-κB pathway activity in the therapeutic study arm (P < 0.04, Fig. 6A). In the prophylactic study group, differences failed to reach statistical significance. Hepatic levels of the NF-κB pathway inhibitory protein, nuclear factor of kappa light polypeptide gene enhancer in B-cells inhibitor alpha (IκBα), were clearly up-regulated in the prophylactic study group and nearly reached statistical significance in the therapeutic study arm (P < 0.01 and P = 0.07 respectively, Fig. 6B), (Fig. 6A,B).

## Discussion

The development and progression of hepatic fibrosis, driven by chronic liver injury and subsequent inflammation, is considered a driving force behind many lethal complications in chronic liver disease, such as liver failure, portal hypertension and hepatocellular carcinoma^25^,^26^,^27^,^28^,^29^,^30^,^31^,^32^,^33^. As the main cellular source for hepatic fibrosis, many attempts have since then focused on HSCs both *in vitro* and in translational animal studies as a target for anti-fibrotic therapy, yet none of them have really made it into clinical practice^7^,^9^,^34^,^35^.

In this study, we demonstrated for the first time that by targeting hepatic inflammation by means of obeticholic acid (OCA), a highly potent and selective first-in-class agonist of the nuclear bile acid receptor FXR, fibrosis can be decreased, both in the process of cirrhogenesis, yet also after cirrhosis has been fully established. This decrease in fibrosis is clinically relevant since it was paralleled by a firm decrease in total intrahepatic vascular resistance (IHVR) and thus portal hypertension. While we have previously shown that acute treatment with OCA decreases IHVR with 16% in TAA cirrhotic rats^16^, following long-term treatment within the same model we were now able to confirm these previous findings and document a further reduction of IHVR up to 25%. This suggests that in the latter now not only the active but also the passive component (i.e. fibrosis) of IHVR is affected. This is further substantiated by the linear correlation between hepatic fibrosis and portal pressure.

Until now, the antifibrotic potential of FXR agonists has proven highly controversial. While Fiorucci *et al.*^12^ and Úbeda *et al.*^36^ reported decreased hepatic fibrosis upon preventive OCA-treatment in porcine serum-induced and CCl_4_–induced liver fibrosis respectively, their findings were contrasted by the data of Fickert *et al.*^15^, finding unaltered effect on fibrosis upon CCl_4_- or S. mansoni-mediated liver injury in animals with genetic FXR-loss. Furthermore, we and others failed to detect functional levels of FXR and FXR pathway activity in isolated HSC *in vitro*^15^,^16^. A further confounding factor in these apparently conflicting data might be the recently revealed beneficial effects of FXR agonists on gut barrier dysfunction, microbiota and bacterial translocation from the gut which in turn might result in reduced liver damage and a secondary decrease in hepatic fibrosis, especially in the CCl4-model^36^,^37^,^38^. In this study, we opted to induce cirrhosis by means of TAA because we have previously shown bacterial translocation to be absent in TAA cirrhotic rats, largely avoiding cross-talk from the gut-liver axis in this manner^38^.

In this study, we showed that FXR activation is quickly lost in HSC upon-culture activation *in vitro* (Supplementary Figure S5) and confirmed that FXR agonists are not able to reactivate the FXR pathway, both in human and murine HSC, independent of a quiescent, activated or stimulated state. Nevertheless, molecular data from liver tissue in our rats clearly suggest decreased HSC activation, with decreased protein levels of alpha-smooth muscle actin (α-SMA) as well as a decreased expression of type I collagen upon OCA treatment. Taken together, these findings imply that the cellular target for FXR-agonism to ultimately decrease hepatic fibrosis in our rat model of toxic cirrhosis is to be searched for further *upstream* in the cascade of HSC activation. For this reason, we isolated different both parenchymal and non-parenchymal hepatic cell types from healthy mouse liver: hepatocytes, Kupffer cells and LSEC, which were exposed to increasing concentrations of OCA *in vitro*, cultured under both stimulated and unstimulated conditions. While we were unable to isolate cells from cirrhotic murine livers, a possible shortcoming in this study, we mimicked the cirrhotic environment by exposing different hepatic cell types to different pro-inflammatory stimuli *in vitro*. Interestingly, we found that OCA dose-dependently inhibited the TNF-α or bacterial endotoxin (LPS)-stimulated expression of MCP-1 in both Kupffer cells and LSEC. This finding is particularly important given the potential for a decrease in MCP-1 to inhibit the chemotactic and fibrogenic potential of HSC and the simultaneous observation of decreased MCP-1 in our OCA-treated rat livers^9^,^39^. The functionality of FXR in the sinusoidal endothelium is in line with our earlier findings, showing strong effects of OCA on HSC hypercontractility, through an equally indirect release of nitric oxide by LSEC^16^. Of further importance, also Kupffer cells, the main cellular *gatekeepers* of hepatic innate immunity were targeted by OCA^40^. In isolated Kupffer cells, this did not only translate in a decrease of pro-inflammatory cytokines such as MCP-1, but also in anti-inflammatory cytokines such as IL-10 (Supplementary Figure S6). Again, this is in line with previous findings exploring the effects of OCA on intestinal immunity in cholestatic rats^38^. The anti-inflammatory effects observed *in vitro* were further again also confirmed in our OCA-treated rat livers, with decreased hepatic necro-inflammation (with a significant decrease in plasma ALT-levels) and an overall decreased expression of crucial pro-inflammation and pro-fibrotic cytokines such as CTGF and TGF-β. Interestingly in our study, also the expression of TIMP-1, a known inhibitor of matrix metalloproteinases secreted amongst others by Kupffer cells, appears decreased upon OCA treatment in cirrhotic rats. In this respect, decreased TIMP-1 suggests an additional increase in extracellular matrix degradation, further ameliorating the existing misbalance between accumulation and degradation of collagens typical of cirrhosis. The finding of decreased TIMP-1 might be of further relevance in this study because it is known to also exert an anti-apoptotic effect on HSC *in vitro*^41^. The suggestion of decreased hepatic cell proliferation and apoptosis is further supported in our study by the finding of decreased hepatic PCNA levels as well as decreased enzymatic degradation of PARP-1, a known substrate of caspase-3, as well as a decreased caspase-3 immunohistochemical staining positivity in OCA treated cirrhotic rats^7^. These findings suggest that the documented resolution of fibrosis observed in cirrhotic rats not only relates to the inhibition of pro-fibrotic signals on HSC as such, but is also critically influencing other determinants of hepatic fibrosis such as ECM degradation, inflammation, proliferation and apoptosis and adds up to the pleiotropic effects of FXR-agonism.

Finally, given the observed overall hepatic anti-inflammatory effect, we focused on the central orchestrator, the NF-κB pathway. This pathway, which is a common pro-inflammatory pathway shared not only by a variety of immune cells but also by endothelial cells and hepatocytes amongst others^42^,^43^,^44^,^45^, is highly involved in the hepatic inflammatory response and subsequent hepatic stellate cell activation^46^,^47^. Previous studies in other settings have shown the relation of FXR to the NF-κB pathway to be one of important mutual antagonism, both at the level of liver and gut^13^,^18^. In our current study, we were able to confirm this decreased hepatic NF-κB activity following FXR agonism also in toxic cirrhotic rats. Moreover, we identified an up-regulation of the inhibitory protein IκBα, which is known to sequester transcription factor NF-κB as an inactive complex in the cytoplasm, as a molecular target of FXR agonist OCA^48^.

Translating the used experimental design into the human situation, this study suggests beneficial effects of OCA in both clinical settings. OCA attenuates inflammation and consequently fibrosis and portal hypertension in ongoing liver injury, such as it might occur in NASH or other intractable liver diseases. On the other side OCA reverses fibrosis and portal hypertension in the situation when the injuring agent was stopped such as it might occur in alcoholic and viral liver cirrhosis. While we have clearly illustrated an anti-inflammatory and subsequent anti-fibrotic effect in our different groups of experimental toxic cirrhosis, some unanswered questions remain. First, while the prophylactic and therapeutic arm of the study show many similarities, there seem to be differences in efficiency and cytokine expression pattern between both groups. Hypothetically, these might relate to the nature of the hepatotoxin used in our study. As such, we chose TAA, rather than CCl_4_, as it is more reflective of the human condition, e.g. alcoholic cirrhosis, since fibrogenesis is much slower in this model and the hepatotoxic insult is usually less severe and accompanied with a mild elevation in aminotransferases^19^,^49^. Secondarily, unlike in the CCl_4_-model, fibrosis did not spontaneously resolve, yet tended to increase further in OCA-untreated rats during 4 weeks after stopping the TAA compound and obtaining cirrhosis. In the *preventive* study, albeit a comparable significant decrease in fibrosis, these latter markers, apart from CTGF and TIMP-1, were less pronounced. A possible explanation is that the ongoing injury (i.e. TAA) antagonizes switch-off of PDGFβ-R, TGF-β and other earlier mentioned markers. On the other hand, the finding of decreased CTGF and TIMP-1 might reflect an initiation of an FXR-mediated antifibrotic effect, yet seem to be hampered in conditions of ongoing TAA intoxication^50^. Finally, we did not assess the role of different cell types in this particular context (such as cholangiocytes, dendritic cells and lymphocytes), which are also known to be involved in hepatic fibrogenesis; yet most of these cell types share the common NF-κB pathway and have been shown to independently respond to FXR agonists in a similar fashion with decreased expression of pro-inflammatory cytokines in previous studies^38^,^43^.

In conclusion, we have shown for the first time that the FXR agonist OCA not only reduces hepatic fibrosis during ongoing cirrhogenesis, it also reverses fibrosis after obtaining cirrhosis in a rat model of toxic cirrhosis, resulting in decreased intrahepatic vascular resistance and improved portal hypertension. Unlike previous reports, this is not mediated by a direct effect on HSC, but rather through indirect effects on immune activation and hepatic cell turn-over, associated with a marked overall decrease in NF-κB activation (through increased IκBα) and cytokines promoting fibrosis and inflammation within the liver parenchyma. *In vitro*, these effects could be reproduced in stimulated Kupffer cells and liver sinusoidal endothelial cells. These findings may in part explain the beneficial effects on hepatic fibrosis in recent phase 3 clinical trials with OCA in patients suffering from PBC and NASH and support extending clinical trials with FXR agonists to other forms of chronic liver injury^51^,^52^.

## Additional Information

**How to cite this article**: Verbeke, L. *et al.* FXR agonist obeticholic acid reduces hepatic inflammation and fibrosis in a rat model of toxic cirrhosis. *Sci. Rep.* **6**, 33453; doi: 10.1038/srep33453 (2016).

## Supplementary Material

Supplementary Information

## Figures and Tables

**Table 1:** Physical and biochemical findings in blood plasma amongst different experimental groups.

	**TAA (n** = **8)**	**TAA** + **OCA (n** = **9)**	**P-value**	**TAA** + **4 (n** = **6)**	**TAA** + **4** + **OCA (n** = **9)**	**P-value**
Body weight (g)	319 ± 11	302 ± 11	0.27	395 ± 9	410 ± 12	0.38
Overall average dose TAA (g/L)	520 ± 13	478 ± 23	0.15	478 ± 19	450 ± 21	0.31
Liver weight	22 ± 2	22 ± 2	0.89	24 ± 2	22 ± 2	0.32
Albumin (g/L)	33.5 ± 0.8	35.1 ± 1.2	0.28	35.2 ± 1.0	36.6 ± 0.7	0.45
AST (IU/L)	85 ± 4	87 ± 7	0.76	100 ± 4	93 ± 4	0.68
ALT (IU/L)	75 ± 4	66 ± 3	0.34	97 ± 4	79 ± 4	*0.01
Bilirubin (mg/dL)	0.3 ± 0.1	0.3 ± 0.2	0.92	0.0 ± 0.0	0.0 ± 0.1	0.90

TAA, thioacetamide; OCA, obeticholic acid; AST, aspartate aminotransferase; ALT, alanine aminotransferase.
