# Supplementary material for: FXR agonist obeticholic acid reduces hepatic inflammation and fibrosis in a rat model of toxic cirrhosis
Source: Sci Rep. 2016 Sep 16;6:33453. doi: 10.1038/srep33453 (PMC5025787; doi:10.1038/srep33453)

FXR agonist obeticholic acid reduces hepatic inflammation and fibrosis in a rat model of toxic cirrhosis

Len Verbeke^1^, Inge Mannaerts^2^, Robert Schierwagen^3^, Olivier Govaere^4^, Sabine Klein^3^, Ingrid Vander Elst^1^, Petra Windmolders^1^, Ricard Farre^5,6^, Mathias Wenes^7,8^,
Massimiliano Mazzone^7,8^, Frederik Nevens^1^, Leo A. van Grunsven^2^, Jonel Trebicka^3^
and Wim Laleman^1^.

**Supplementary files**

**Supplementary Figure S1.**

**Schematic representation of experimental groups.**


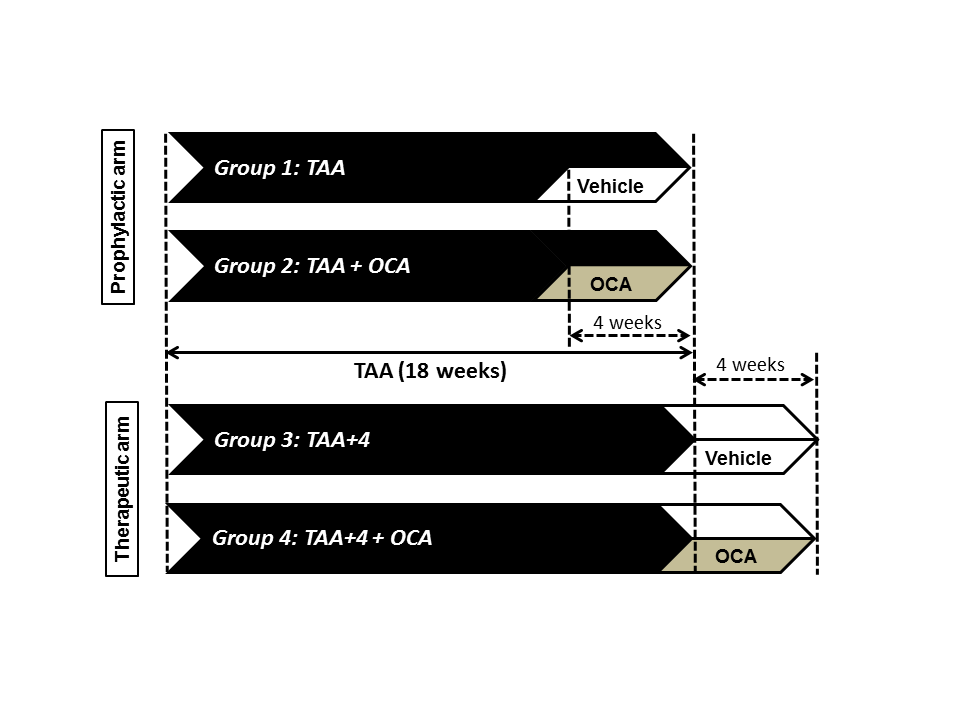


**Supplementary Methods S2.**


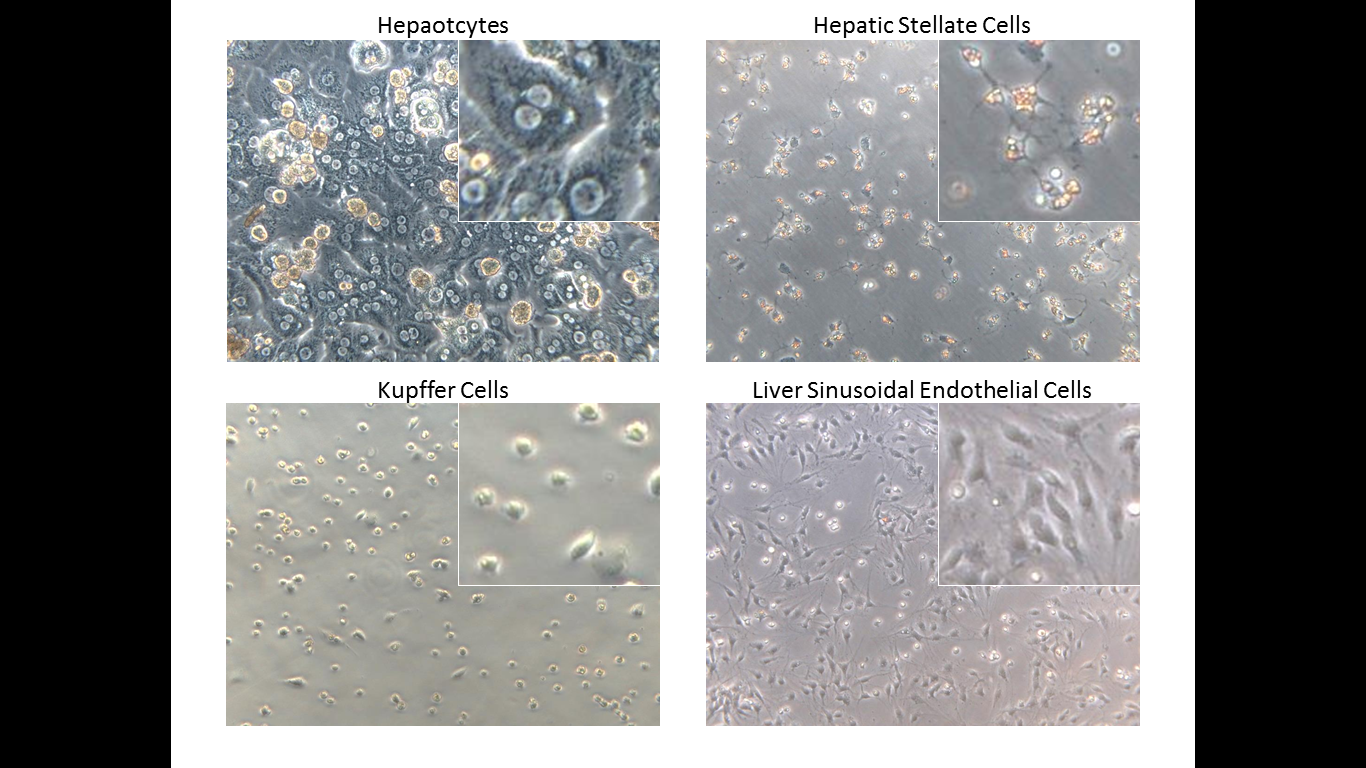

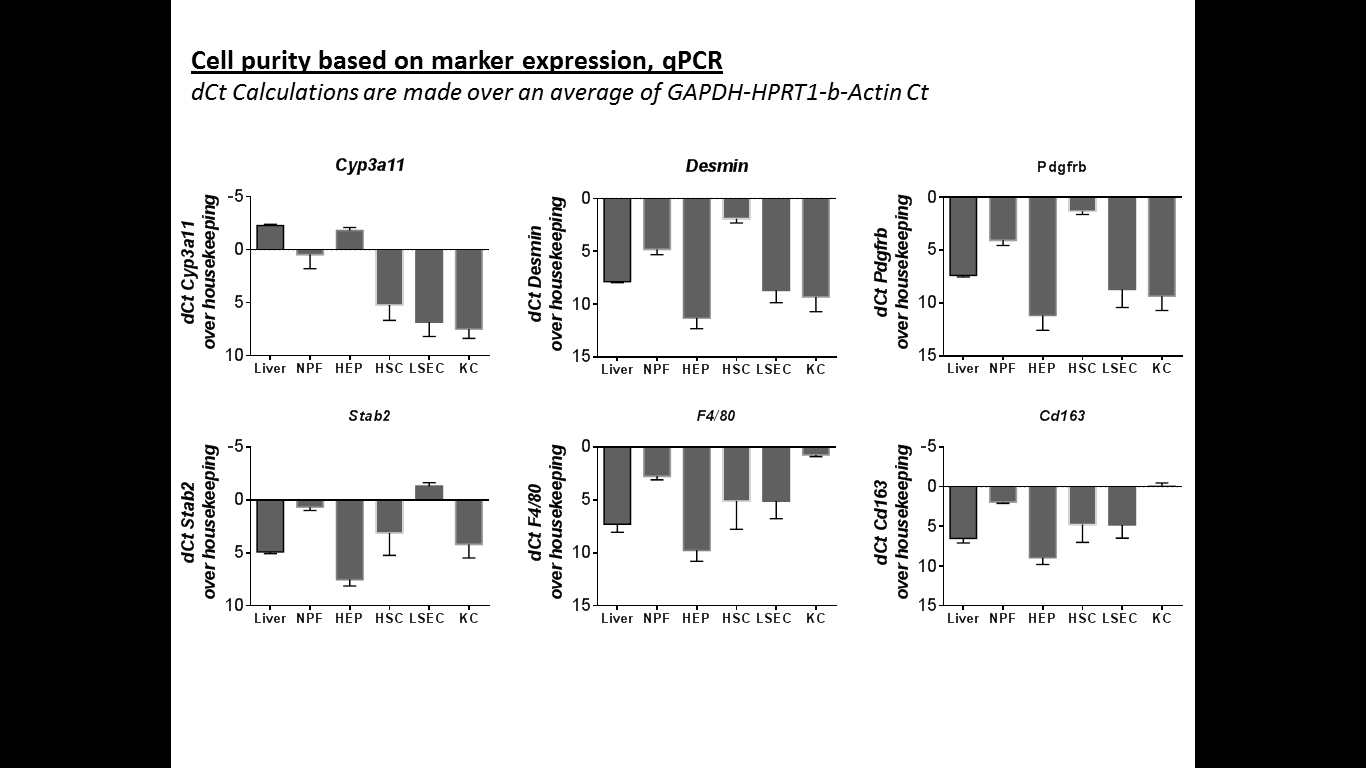


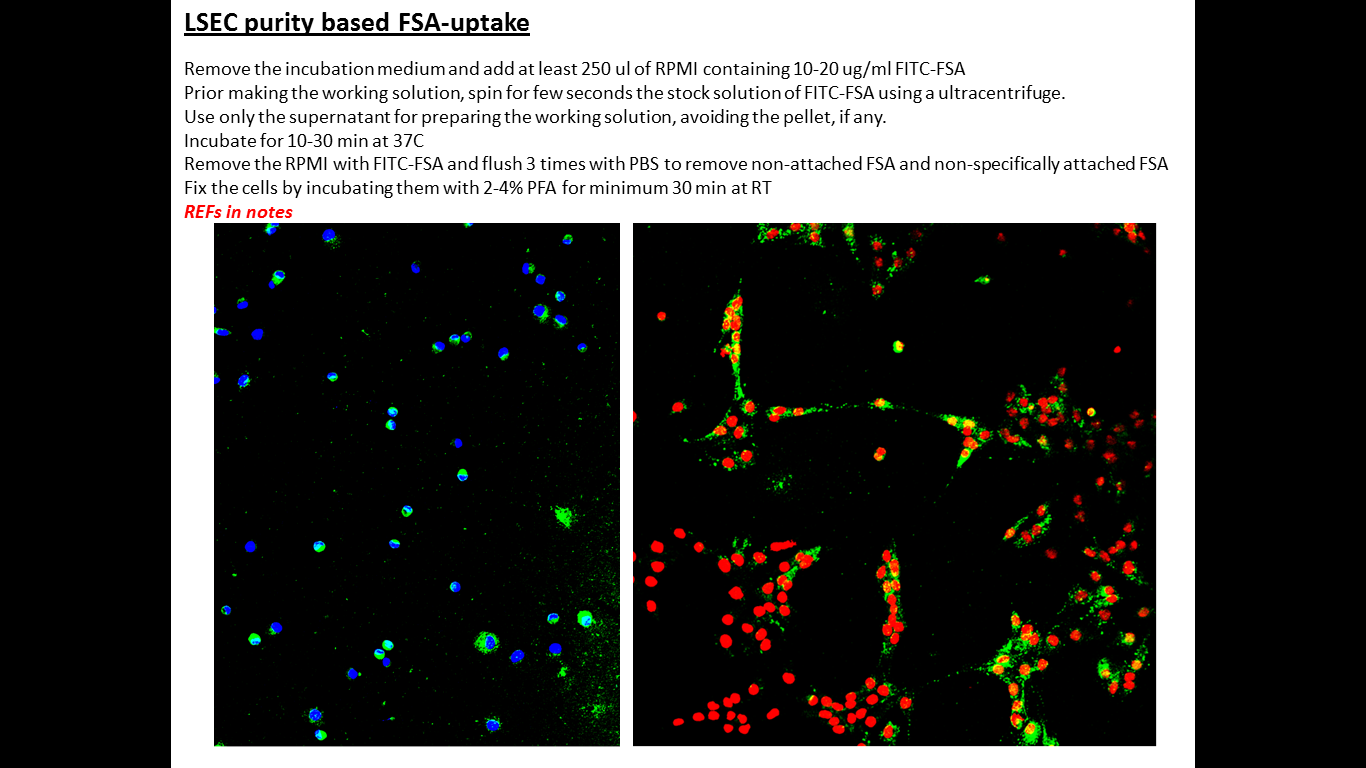


**Funtional assay for LSEC based on FITC-labeled serum albumin uptake by LSEC scavenger receptors**

**
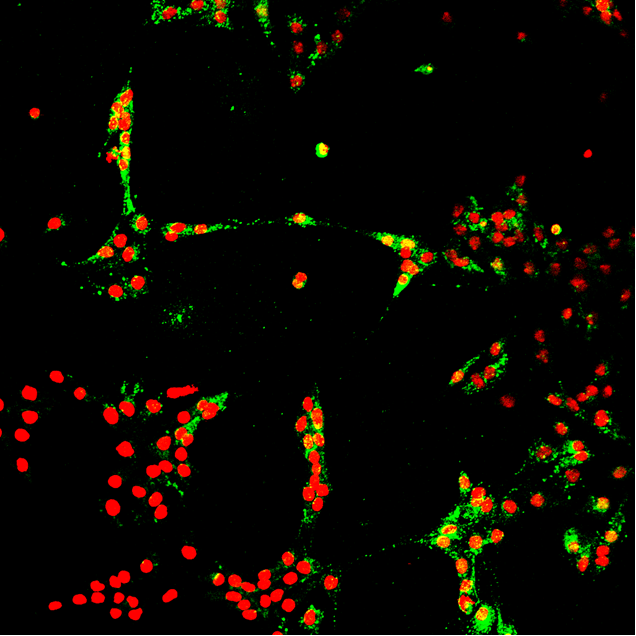

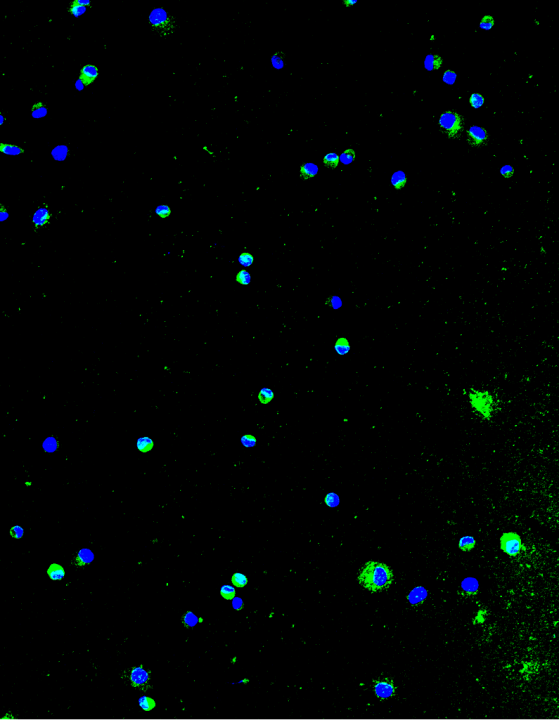
**

Remove the incubation medium and add at least 250 ul of RPMI containing 10-20 ug/ml FITC-FSA. Prior making the working solution, spin for few seconds the stock solution of FITC-FSA using a ultracentrifuge. Use only the supernatant for preparing the working solution, avoiding the pellet, if any. Incubate for 10-30 min at 37C. Remove the RPMI with FITC-FSA and flush 3 times with PBS to remove non-attached FSA and non-specifically attached FSA. Fix the cells by incubating them with 2-4% PFA for minimum 30 min at RT

*Ref.*

*[1] G. Xie, L. Wang, X. Wang, L.D. DeLeve, Isolation of periportal, midlobular, and centrilobular rat liver sinusoidal endothelial cells enables study of zonated drug toxicity, Am J Physiol Gastrointest Liver Physiol, 299 (2010) G1204-1210.*

*[1] W. Eskild, B. Smedsrod, T. Berg, Receptor mediated endocytosis of formaldehyde treated albumin, yeast invertase and chondroitin sulfate in suspensions of rat liver endothelial cells, Int J Biochem, 18 (1986) 647-651.*

*[2] K.H. Elvevold, G.I. Nedredal, A. Revhaug, B. Smedsrod, Scavenger properties of cultivated pig liver endothelial cells, Comp Hepatol, 3 (2004) 4.*

*[3] W. Eskild, G.M. Kindberg, B. Smedsrod, R. Blomhoff, K.R. Norum, T. Berg, Intracellular transport of formaldehyde-treated serum albumin in liver endothelial cells after uptake via scavenger receptors, Biochem J, 258 (1989) 511-520.*

**Supplementary Methods S3.**

***RT-PCR***

The relative expression of different genes was assessed by RT-PCR. For this purpose, total RNA was extracted from liver samples and cultured isolated cells (n=5-8 per group) in TRIzol reagent (Invitrogen, Belgium) and reverse transcribed. Complementary DNA was amplified by PCR by use of rat-specific primers and probes in a ready-to-use mix (Life technologies, Germany) or by gene-specific primers produced by Integrated DNA Technologies (Leuven). RT-PCR (ABI 7300 sequence detector) and PCR reaction were performed as described. Samples were normalized to the housekeeping gene glyceraldehyde-3-P-dehydrogenase (GADPH) and fold-change expression was compared to the comparative vehicle-treated control groups using the 2-ΔΔCT method, as previously described.

|  | Forward | Reverse |
| --- | --- | --- |
| **Col1a1** | acctaagggtaccgctgga | tccagcttctccatctttgc |
| **α-SMA** | ccagcaccatgaagatcaag | tggaaggtagacagcgaagc |
| **TGF-β1** | tggagcaacatgtggaactc | gtcagcagccggttacca |
| **PDGF-βR** | tgcagagacctcaaaaggtg | cctgatcttcctcccagaaa |
| **CTGF** | tgacctggaggaaaacattaaga | agccctgtatgtcttcacactg |
| **F4/80** | cctggacgaatcctgtgaag | ggtgggaccacagagagttg |
| **MCP-1** | catccacgtgttggctca | gatcatcttgctggtgaatgagt |
| **TIMP-1** | gcaactcggacctggtcataa | cggcccgtgatgagaaact |
| **IFN-Υ** | atctggaggaactggcaaaa | ttcaagacttcaaagagtctgagg |
| **FXR** | caaaatgactcaggaggagtacg | tccttgatgtattgtctgtctgg |
| **SHP** | tgggtcccaaggagtatgc | gctccaagacttcacacagtg |
| **IL-10** | cagagccacatgctcctaga | gtccagctggtcctttgttt |
| **IL-6** | gctaccaaactggatataatcagga | ccaggtagctatggtactccagaa |
| **PDGFA** | caccagcagcgtcaagtg | ttcctgacatactccactttgg |
| **PDGFB** | cggcctgtgactagaagtcc | gagcttgaggcgtcttgg |
| **MMP-13** | tgtttgcagagcactacttgaa | cagtcacctctaagccaaagaaa |

Col1a1, collagen type 1; α-SMA, alpha-smooth muscle actin; TFG-β1, transforming growth factor-beta 1; PDGF-βR, platelet-derived growth factor-beta receptor; CTGF, connective tissue growth factor; MCP-1, monocyte chemo-attractant protein-1; TIMP-1, tissue inhibitor of metallopeptidase-1; IFN-Υ, interferon-gamma; FXR; farnesoid-X receptor; SHP, small heterodimer partner; IL-1,-6,-10 Interleukin-1,-6,-10; PDGFA/B, platelet-derived growth factor A/B; MMP-13: matrix metallopeptidase-13.

|  | Assay ID |
| --- | --- |
| **Col1a1** | Rn00801649_g1 |
| **α-SMA** | Rn01759928_g1 |
| **TGF-β1** | Rn00572010_m1 |
| **PDGF-βR** | Rn00709573_m1 |
| **CTGF** | Rn00573960_g1 |
| **F4/80** | Rn01527631_m1 |
| **MCP-1** | Rn00580555_m1 |
| **TIMP-1** | Rn00587558_m1 |

Col1a1, collagen type 1; α-SMA, alpha-smooth muscle actin; TFG-β1, transforming growth factor-beta 1; PDGF-βR, platelet-derived growth factor-beta receptor; CTGF, connective tissue growth factor; MCP-1, monocyte chemo-attractant protein-1; TIMP-1, Tissue inhibitor of metallopeptidase-1.

**Supplementary Methods S4.**

***Western blot***

Snap-frozen liver samples were homogenized in a buffer containing 25 mM Tris/HCl, 5 mM ethylenediamine tetraacetic acid, 1 mM benzamidine, 10 µM phenylmethanesulfonyl fluoride and 10 µg/ml leukopeptin. Protein determination was performed by use of a Dc-Assay kit (Biorad, Germany). Samples were subjected to sodium dodecyl sulfate polyacrylamide electrophoresis (10% gels) and proteins were blotted on nitrocellulose membranes. The membranes were blocked and incubated with the primary antibody for α-SMA (Abcam, UK), proliferating cell nuclear antigen (PCNA, Santa Cruz, USA), poly ADP ribose polymerase-1 (PARP-1, Becton Dickinson, Germany), while GADPH (Santa Cruz, USA) served as an internal control. Thereafter, the membranes were incubated with the corresponding secondary peroxidase-coupled Abs (Calbiochem). After enhanced chemiluminescence (Amersham, UK), digital detection was evaluated using Chemi-Smart technology (PeqLab Biotechnologie GmbH, Germany). For IκBα assessment, a liver specimen of about 100mg was lysed in 1 ml RIPA buffer (50mM Tris pH8, 150mM NaCl, 0,5% SDS, 1% triton, 5mM EDTA, 0,5% Na-deoxycholate) containing protease and phosphatase inhibitors (Roche, Belgium), and using Lysing matrix D (MP-biomedicals, USA) and a Ribolyser (Bio-rad, Belgium) 3 x 60s at maximum speed. Protein concentration was determined using the Pierce BCA Protein Assay Kit (Thermo Scientific, USA). The following antibodies were used: IκBα (Cell Signaling), and β-tubulin (Abcam), and appropriate HRP-conjugated secondary antibodies (Santa Cruz). Signal was visualized by Enhanced Chemiluminescent Reagents (ECL, Invitrogen) or West Femto by Thermo Scientific according to the manufacturer’s instructions and acquired by a LAS 4000 CCD camera with ImageQuant software (GE Healthcare).

**Supplementary Figure S5.**

**FXR and SHP mRNA expression data in different isolated murine hepatic cell types.**


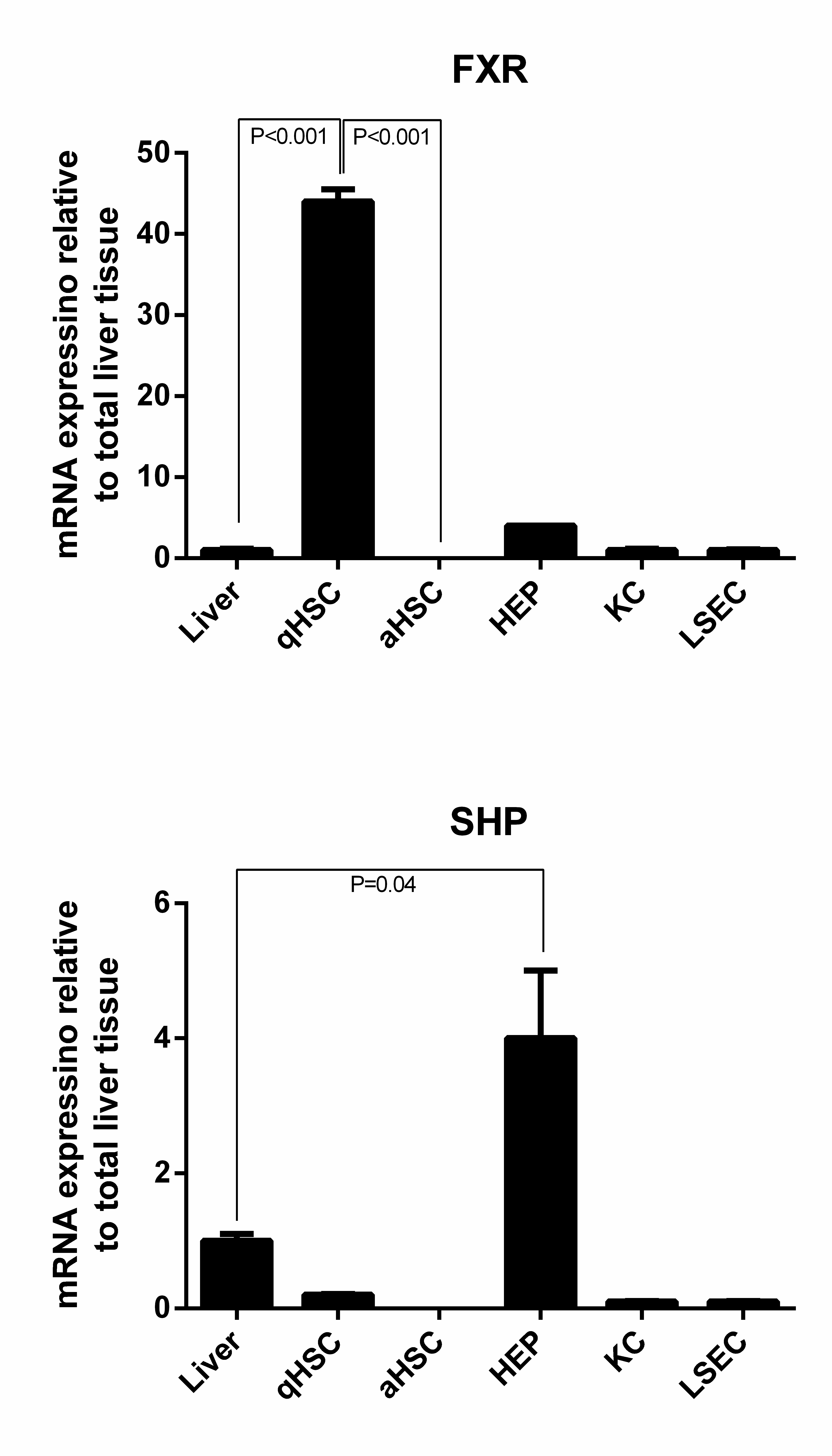


**Supplementary Figure S6.**

**IL-10 mRNA expression in isolated Murine Kupffer cells, in vitro stimulated with OCA +/- TNF-α +/- LPS.**


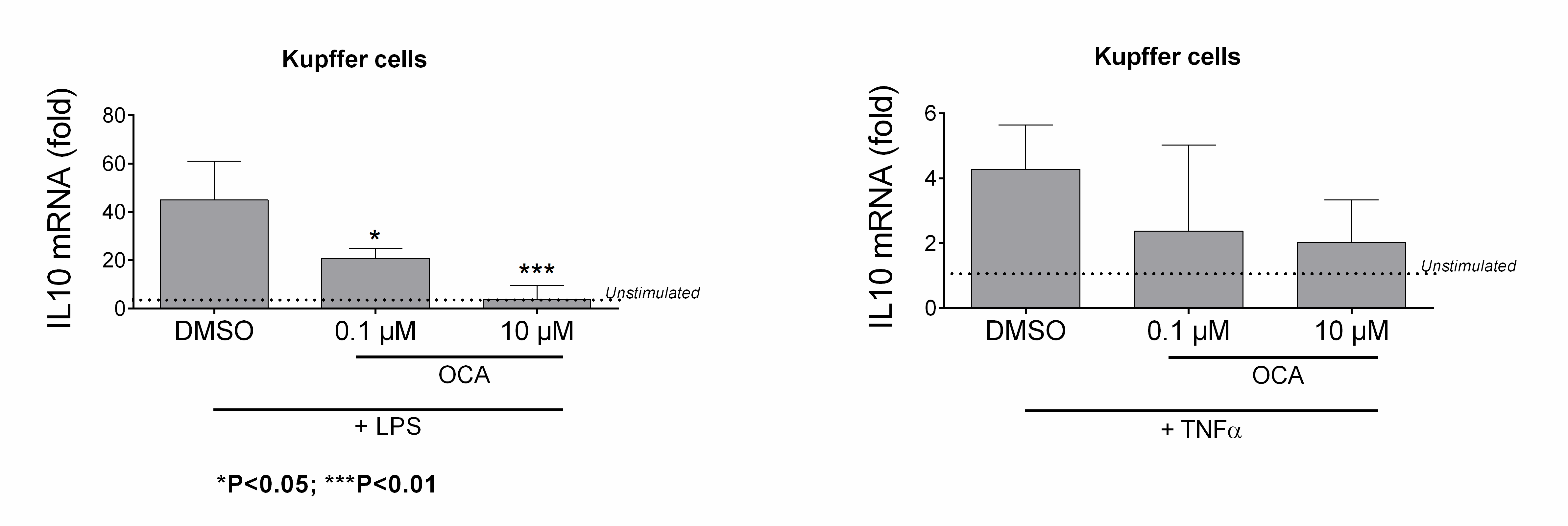

Supplement: Supplementary Information [file srep33453-s1.doc]
